# Indirect visualization of endogenous nuclear actin by correlative light and electron microscopy (CLEM) using an actin-directed chromobody

**DOI:** 10.1007/s00418-019-01795-3

**Published:** 2019-06-01

**Authors:** Mohamed E. A. Abdellatif, Lisa Hipp, Matthias Plessner, Paul Walther, Bernd Knöll

**Affiliations:** 1 Central Facility for Electron Microscopy, Ulm University, Albert-Einstein-Allee 11, 89081 Ulm, Germany; 2 Institute of Physiological Chemistry, Ulm University, Albert-Einstein-Allee 11, 89081 Ulm, Germany; 3 Institute of Experimental and Clinical Pharmacology and Toxicology, University of Freiburg, Albertstr. 25, 79104 Freiburg, Germany

**Keywords:** Nucleus, Actin, Immunogold, EM, Nanobody, Neurodegeneration

## Abstract

Actin fulfills important cytoplasmic but also nuclear functions in eukaryotic cells. In the nucleus, actin modulates gene expression and chromatin remodeling. Monomeric (G-actin) and polymerized actin (F-actin) have been analyzed by fluorescence microscopy in the nucleus; however, the resolution at the ultrastructural level has not been investigated in great detail. We provide a first documentation of nuclear actin in mouse fibroblasts by electron microscopy (EM). For this, we employed correlative light and electron microscopy on the same section using actin-directed nanobodies recognizing endogenous monomeric and polymeric actin proteins (so-called nuclear Actin-chromobody-GFP; nAC-GFP). Indeed, using this strategy, we could identify actin proteins present in the nucleus. Here, immunogold-labeled actin proteins were spread throughout the entire nucleoplasm. Of note, nuclear actin was complementarily localized to DAPI-positive areas, the latter marking preferentially transcriptionally inactive heterochromatin. Since actin aggregates in rod structures upon cell stress including neurodegeneration, we analyzed nuclear actin at the ultrastructural level after DMSO or UV-mediated cell damage. In those cells the ratio between cytoplasmic and nuclear gold-labeled actin proteins was altered compared to untreated control cells. In summary, this EM analysis (i) confirmed the presence of endogenous nuclear actin at ultrastructural resolution, (ii) revealed the actin abundance in less chromatin-dense regions potentially reflecting more transcriptionally active euchromatin rather than transcriptionally inactive heterochromatin and (iii) showed an altered abundance of actin-associated gold particles upon cell stress.

**Electronic supplementary material:**

The online version of this article (10.1007/s00418-019-01795-3) contains supplementary material, which is available to authorized users.

## Introduction

Actin fulfills functions not only in the cytoplasm but is now widely accepted to play a role in the nucleus of cells (Bajusz et al. 2018; Melak et al. 2017; Virtanen and Vartiainen 2017). Globular (G-) actin monomers modulate the general RNA transcription machinery and interact with several chromatin-remodeling complexes. Furthermore, nuclear G-actin controls specific gene-expression programs exerted by MRTF (myocardin-related transcription factor) transcription factors, cofactors of the serum response factor (SRF) (Vartiainen et al. 2007). The presence of polymerized filamentous actin (F-actin) in the nucleus was more disputed. However, recent data have shown transient nuclear F-actin assembly stimulated by integrin-mediated cell adhesion (Plessner et al. 2015), serum application (Baarlink et al. 2013) and mitotic exit of cells (Baarlink et al. 2017). First nuclear F-actin functions have been ascribed including heterochromatin repair (Caridi et al. 2018) and nuclear re-organization of cells after mitotic exit (Baarlink et al. 2017). Furthermore, persisting microfilaments might interfere with RNA polymerase II transcription (Serebryannyy et al. 2016a). Beside these physiological processes, cell damage and stress including heat shock, DNA damage by UV irradiation and DMSO (dimethyl sulfoxide) trigger nuclear F-actin polymerization (Belin et al. 2015; Serebryannyy et al. 2016b). In several neurodegenerative diseases, such F-actin filaments form aggregates so-called rods together with the actin-binding protein cofilin (Bamburg et al. 2010; Munsie and Truant 2012; Serebryannyy et al. 2016b).

Investigation of nuclear F-actin was strongly hampered by the failure of phalloidin, the prototypical marker of F-actin, to label microfilaments robustly in the nucleus. Recently, several new actin-detecting probes including LifeAct, Utrophin and actin-directed nanobodies (nuclear Actin-chromobody-GFP; referred to as nAC-GFP) were employed to visualize endogenous nuclear F-actin with fluorescence microscopy (Spracklen et al. 2014; Riedl et al. 2008; Plessner et al. 2015; Melak et al. 2017; Belin and Mullins 2013). For instance, nAC-GFP, a fusion protein of an actin-directed nanobody tagged C-terminally with GFP and an NLS (nuclear localization signal) revealed actin microfilament formation during serum stimulation, cell adhesion and mitosis (Baarlink et al. 2013, 2017; Plessner et al. 2015). This nanobody does not affect the equilibrium between cytoplasmic and nuclear actin and stable expression does not induce F-actin formation in quiescent cells (Plessner et al. 2015). Notably, nAC-GFP recognizes monomeric as well as filamentous actin (Melak et al. 2017). This Actin-Chromobody has been successfully applied to specifically label actin in mammalian cell nuclei (Plessner et al. 2015), in tobacco leaf cells (Rocchetti et al. 2014) and in zebrafish (Panza et al. 2015). So far, unspecific detection of other cellular proteins by this chromobody has not been reported. Nonetheless, currently, this chromobody represents a novel but rather unexplored tool.

So far, localization of endogenous nuclear actin was comprehensively analyzed at the immunofluorescence level, but ultrastructural analysis with EM is missing with few exceptions (Parfenov et al. 1995; Kysela et al. 2005; Hofmann et al. 2004). Here, co-localization of RNA Polymerase II with nuclear actin was demonstrated. In previous EM studies we showed localization of overexpressed actin mutant proteins in F-actin structures in the nucleus (Kokai et al. 2014; Stern et al. 2009). This was further confirmed by immunofluoresence imaging of fusion proteins of wildtype actin and fluorescent proteins (Kalendova et al. 2014). These studies demonstrated that different cell types have the ability to polymerize actin filaments in the nucleus if a critical nuclear abundance of actin proteins is reached. However, in those studies we achieved high levels of overexpression and nuclear localization of actin mutant proteins through fusion of actin proteins with a NLS sequence normally not present in actin. Thus, actin levels were artificially elevated and thereby exceeded endogenous actin levels normally present in the nucleus. Therefore, in the current study investigating endogenous actin levels, detection of actin filaments should be more difficult compared to our previous studies using ectopic actin protein overexpression (Kokai et al. 2014; Stern et al. 2009).

In the present study we chose an indirect approach to analyze such endogenous nuclear actin. We used the nAC-GFP nanobodies consisting of heavy-chain-only antibodies with a molecular mass of only around 13 kDa and a much smaller size (2 nm × 4 nm) in comparison to conventional antibodies. This nAC-GFP chromobody (see above) was employed to analyze endogenous actin structures and actin sub-nuclear distribution in fibroblasts at EM level. For this, a correlative light and electron microscopy (CLEM) protocol was established allowing for simultaneous localization of fluorescently-labeled actin molecules (in the light microscope) and immunogold-labeled actin molecules [in the electron microscope; (Roth et al. 1978)] on one section of the same cell. CLEM is a useful versatile approach to localize different macromolecules of interest as shown before (Fabig et al. 2012; Kukulski et al. 2011, 2012; Lysova et al. 2018; Schwarz and Humbel 2014).

## Materials and methods

### Cell culture

Cells were cultured on 3-mm carbon-coated sapphire discs (160 µm thick; Engineering Office M. Wohlwend GmbH, Switzerland). Sapphire discs were glow-discharged (Edwards High vacuum Glow discharge device, Germany), UV-sterilized (UVP UV crosslinker, UK) and placed on 12-mm glass coverslips. The discs and coverslips were coated with fibronectin at 40 µg/ml in 0.1% gelatine for 15 min at 37 °C. NIH3T3 cells (30,000–40,000 cells) were seeded on top of the sapphire discs and coverslips. The next day, cells were transfected with the nAC-GFP-NLS construct (Plessner et al. 2015) using Lipofectamine 2000 (Invitrogen) in OptiMEM at 1.8 ng/µL final concentration. After 6 h, OptiMEM was replaced by DMEM/10% FCS and cells were kept for 1 day at 37 °C/5% CO_2_ until high-pressure freezing.

### High-pressure freezing and freeze substitution

Before freezing, nAC-GFP expression was inspected in living cells using the Zeiss Observer Z1 Fluorescence Microscope (Zeiss, Germany). Control cells were immediately frozen without additional treatment. For stress induction, cells were incubated with 10% DMSO in medium for 30 min in the incubator. For UV damage, we applied 50 J/m^2^ in an UV crosslinker. Subsequently, cells were washed 1 × with PBS and cells were allowed to recover for 6 h in medium before freezing. For high-pressure freezing, a 50 µm-thick gold spacer ring with a 2 mm central bore (Plano, Germany) was sparingly dipped in 1-hexadecene and mounted between two 3-mm sapphire discs harboring the cells (with the cells facing inwards to the gold ring) as reported before (Hawes et al. 2007). This sandwich was tightly sealed inside the high-pressure freezer holder to avoid trapping of air bubbles inside. It was then quickly inserted in the Wohlwend HPF Compact 01 high-pressure freezer (Wohlwend GmbH, Switzerland) and was high-pressure frozen. The holder was quickly removed from the high-pressure freezing chamber and inserted into liquid nitrogen to transfer the sapphire discs into BEEM^®^ capsules (VWR, Germany) for further storage in liquid nitrogen until freeze substitution. The freeze substitution media consisted of 0.1% uranyl acetate and 5% water in acetone but devoid of osmium tetroxide that would otherwise lead to the potential loss or masking of antigenicity (Newman and Hobot 1999). Freeze substitution was performed over a 17 h-period with a gradual temperature increase from − 87  to − 20 °C using a custom-made computer-controlled substitution apparatus. On the following day, unbound uranyl acetate was removed by 3 × 1 h isopropanol washing (acetone was avoided because its traces negatively affect the resin polymerization) while maintaining − 20 °C temperature.

### Sample embedding

The acrylic resin LR gold (London Resin Company LTD, England) was used for embedding. LR gold polymerization was initiated by benzyl and benzoyl peroxide at a concentration of 0.1% (*w*/*w*) in LR gold. The solution was mixed thoroughly by ultra-sonication before cooling down to − 20 °C. For sample embedding, the solution was mixed with isopropanol in a stepwise fashion (25% LR gold, 50% LR gold, 75% LR gold, and 100% LR gold) and samples were incubated for 1 h each. Afterwards, samples were transferred into fresh LR gold making sure to exclude oxygen from the Eppendorf tubes. This was important to avoid termination of the radical chain reaction required for LR gold polymerization (curing). The samples were then placed in a metal rack inside a specialized tank with UV emission lamps at − 20 °C for LR gold curing with UV light. They were left to gradually warm up to room temperature before the UV light source was switched off after 2 days and the resin was completely polymerized.

### Sectioning

The LR gold blocks were broken off by dipping the tip of the block into liquid nitrogen. This led to the detachment of the tip with the sapphire disc leaving the cells covered with the carbon layer on the rest of the block. The blocks were then mounted in a Leica Ultracut UCT ultramicrotome (Leica Microsystems, Wetzlar) and cut into 100 nm thick sections with an ultra 45° diamond knife (Diatome, Biel, Switzerland). The sections were picked up onto 200 mesh copper finder grids with support Formvar films (Plano, Germany). They were left to dry at room temperature before being further processed for immunolabeling.

### Immunolabeling

Immunolabeling was performed on the section by floating the grids (section side down) on droplets of the labeling reagents that were pipetted on Parafilm^®^ M (Pechiney Plastic Packaging, WI, USA) inside a humid chamber. The reagents were quickly spun to get rid of solid contaminants. For blocking we used PBS/1% cold water fish skin (CWFS) gelatin/0.5% BSA/1% skim milk based on Fabig et al. (2012) and Schwarz and Humbel (2014). After incubation with the blocking buffer for 5 min, the primary antibody was applied: goat anti-GFP (Rockland, Germany; final 5 µg/ml) diluted in blocking buffer for 2 h at room temperature. After 6 × 2 min washing in blocking buffer, grids were incubated with the colloidal gold-conjugated secondary antibody: rabbit anti-goat IgG (H + L), 6 nm gold (Aurion, the Netherlands) at a concentration of 1:20 in blocking buffer for 1 h. After 3 × 2 min washing with blocking buffer and 3 × 2 min PBS washing, grids were incubated with the fluorescent secondary antibody: Cy3-conjugated AffiniPure rabbit-anti-goat IgG (H + L; Jackson ImmunoResearch, Germany; 5 µg/ml) in blocking buffer for 1 h in the dark. Later on the Cy3 fluorescence was converted to green color using Axiovision software (Zeiss). Subsequently, grids were washed 3 × 2 min with blocking buffer and 3 × 2 min with PBS. Counterstaining was done using 4′, 6-diamidino-2-phenylindole (DAPI; 0.1 µg/ml in water) for 10 min. The grids were washed with drops of bidistilled water before they were mounted between glass slides and coverslips with the help of 50% glycerol (VWR, Germany) and 1% n-propyl gallate (AppliChem, Germany) in bidistilled water and sealed with duplicating silicone (Picodent twinsil, Germany). In a control experiment (Fig. 2f), to investigate nonspecific aggregations of gold particles, we applied 20-fold diluted 6 nm gold-conjugated antibodies to Formvar-coated copper grids (treated with poly-l-lysine) with no subsequent washing steps to provoke a nonspecific binding. For labeling of double-strand breaks we applied rabbit anti-*γ*-H2AX (Abcam AB2893; 1:200) antibodies according to standard immunocytochemistry protocols.

### Fluorescence and electron microscopy

Samples were imaged with a 100× oil-objective at a Zeiss Axiovert 200 M fluorescence microscope (Zeiss, Germany). Regions of interests (ROIs) with cells showing nuclear fluorescent signals were imaged and the localization on the coordination grid noted. The labeled grids were gently un-mounted from the glass slides and cover-slipped with water and left to dry at room temperature. The specimen grids were then coated with 5 nm-thick carbon using the BAF 300 (Balzers, Germany) and inserted in the JEM-2100 (Jeol, Japan). The ROIs (previously selected via fluorescence microscopy) were located with the help of the finder grid orientation and 1024 × 1024 (pixel size: 1.94 nm) images of entire cell nuclei in the ROI were recorded using the dark-field detectors to resolve nanogold particles.

### Image processing

For each cell of interest consecutive images of the entire nucleus were acquired. The images were stitched together using the Stitching plug-in (Preibisch et al. 2009) in Fiji (Schindelin et al. 2012). Full-resolution images of all mosaic pictures (Figs. 2a, 3a and 4d) are provided in the Supplement. The gold particles were identified by applying a mask only recognizing gold particles and quantified using the “Threshold” and “Analyze particles” functions of Fiji. For cluster analysis of the gold particles, the Fiji plugin BioVoxxel was used (Healy et al. 2018) employing the “2D particle distribution” macro to investigate the significance of clustering and the “neighbor analysis” macro to quantify cluster sizes by counting how many neighbors one individual particle has in a certain distance. Particles within a distance of 29 nm were still classified as neighbors. False-color labeling of the gold particles for visualization purposes was done after cluster analysis. Thereby gold particles were magnified to 90 nm to enable visibility of the gold particle structure in the overlay with the fluorescent image of the whole cell.

## Results and discussion

### Establishing a protocol for correlative light and electron microscopy (CLEM)

In this study we analyzed endogenous nuclear actin with EM in NIH3T3 mouse embryonic fibroblasts. First of all, we established a correlative light and electron microscopy staining protocol. We adopted a CLEM approach based on several published protocols to best suit our aim to morphologically characterize nuclear actin (Fabig et al. 2012; Kukulski et al. 2011, 2012; Lysova et al. 2018; Schwarz and Humbel 2014). First, the sample preparation through high-pressure freezing, freeze substitution and low-temperature embedding in LR gold guaranteed a high level of ultrastructure preservation while maintaining antigenicity. Second, smaller gold particles lead to a higher labeling density attributed to the reduction in the steric hindrance (Mayhew 2011; Stierhof et al. 1991). Hence, we used the 6 nm nanogold-conjugated secondary antibody during immunolabeling. Third, one of the best contrasts for gold markers can be achieved in 200 kV STEM (Scanning Transmission Electron Microscopy) with the high-angle annular dark-field (HAADF) detectors (Sousa et al. 2007). Indeed, we were able to directly visualize the gold particles in STEM using the dark-field detector with a high spatial resolution without the need for silver enhancement.

**Fig. 1:**
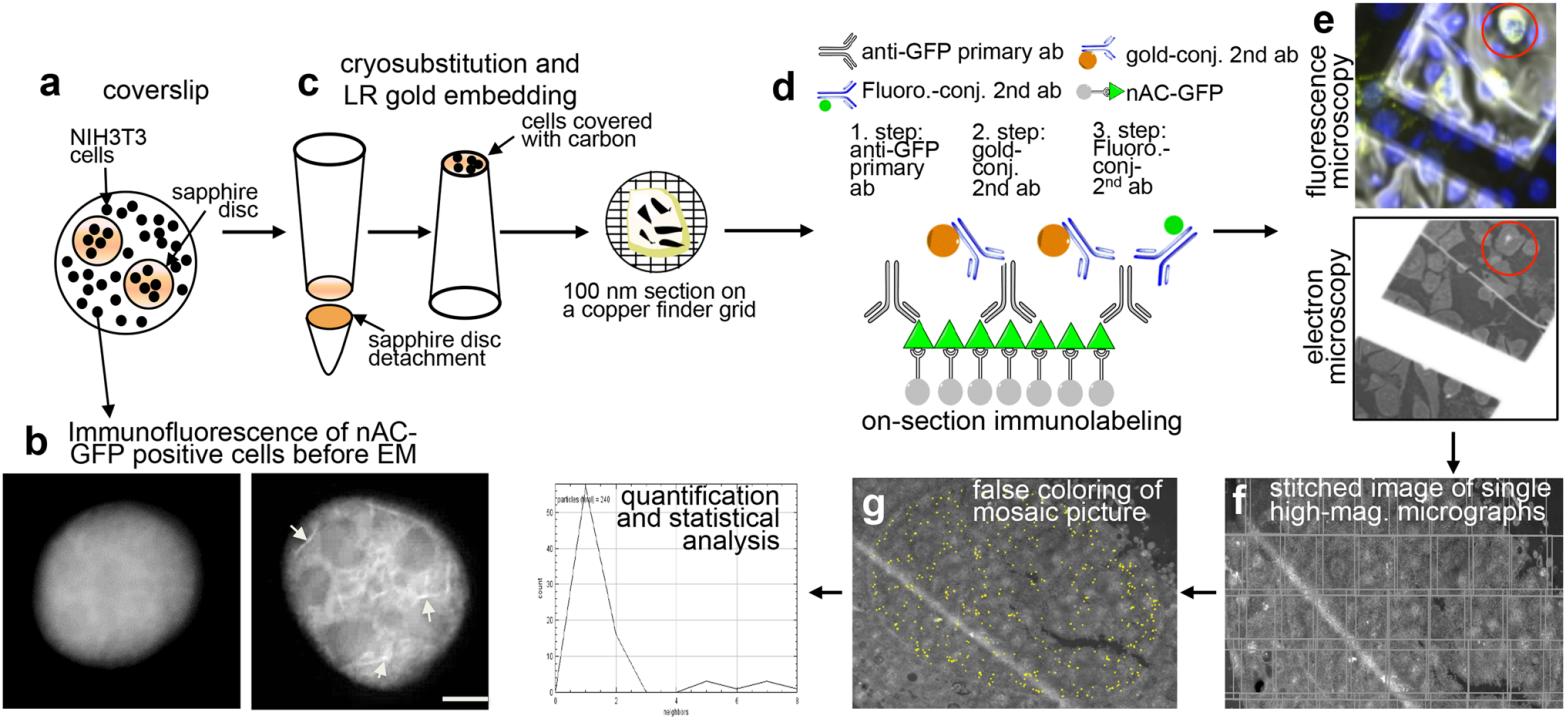
Correlative light and electron microscopy approach. **a** NIH3T3 cells were spread on sapphire discs placed on coverslips and transfected with the nAC-GFP construct. **b** Examples of GFP signal of cells expressing nAC-GFP in the nucleus. Arrows point at filamentous structures. **c** Cells were cryo-fixed by high-pressure freezing, freeze substituted and LR-gold embedded, followed by cutting 100 nm sections that were picked up onto copper grids. **d** Staining was performed with anti-GFP-directed antibodies recognizing the GFP-tag of the chromobody nAC-GFP. Two different secondary antibodies, conjugated with either 6 nm gold particles or fluorophores were applied. **e** Fluorescent cells (red circle) were marked on the grids to allow re-examination of the same cell by EM. **f** A mosaic of single high-resolution EM pictures was prepared from a section. **g** Gold particles were false-color-labeled in yellow. Scale-bar **b** = 2 μm

Cells were seeded on coverslips with sapphire discs on top allowing cells to adhere to either the coverslip or disc (Fig. 1a). The coverslip allows for analyzing the same batch of cells by light microscopy (Fig. 1b) before proceeding with the labor-intensive EM preparation of sapphire discs. After cryo-immobilization by high-pressure freezing, LR gold-embedded cells were sectioned and 100 nm sections were transferred on copper grids (Fig. 1c). To visualize endogenous actin, cells expressed a GFP-tagged actin-directed nanobody localizing to the nucleus via an NLS (Plessner et al. 2015). nAC-GFP was chosen since it appears to not impinge on endogenous actin dynamics as severely as other actin probes at high expression levels (Melak et al. 2017). Furthermore, the specificity of this chromobody for actin structures was confirmed in several species including several mammalian cell types and plant cells (Rocchetti et al. 2014; Plessner et al. 2015; Panza et al. 2015; Melak et al. 2017).

**Fig. 2:**
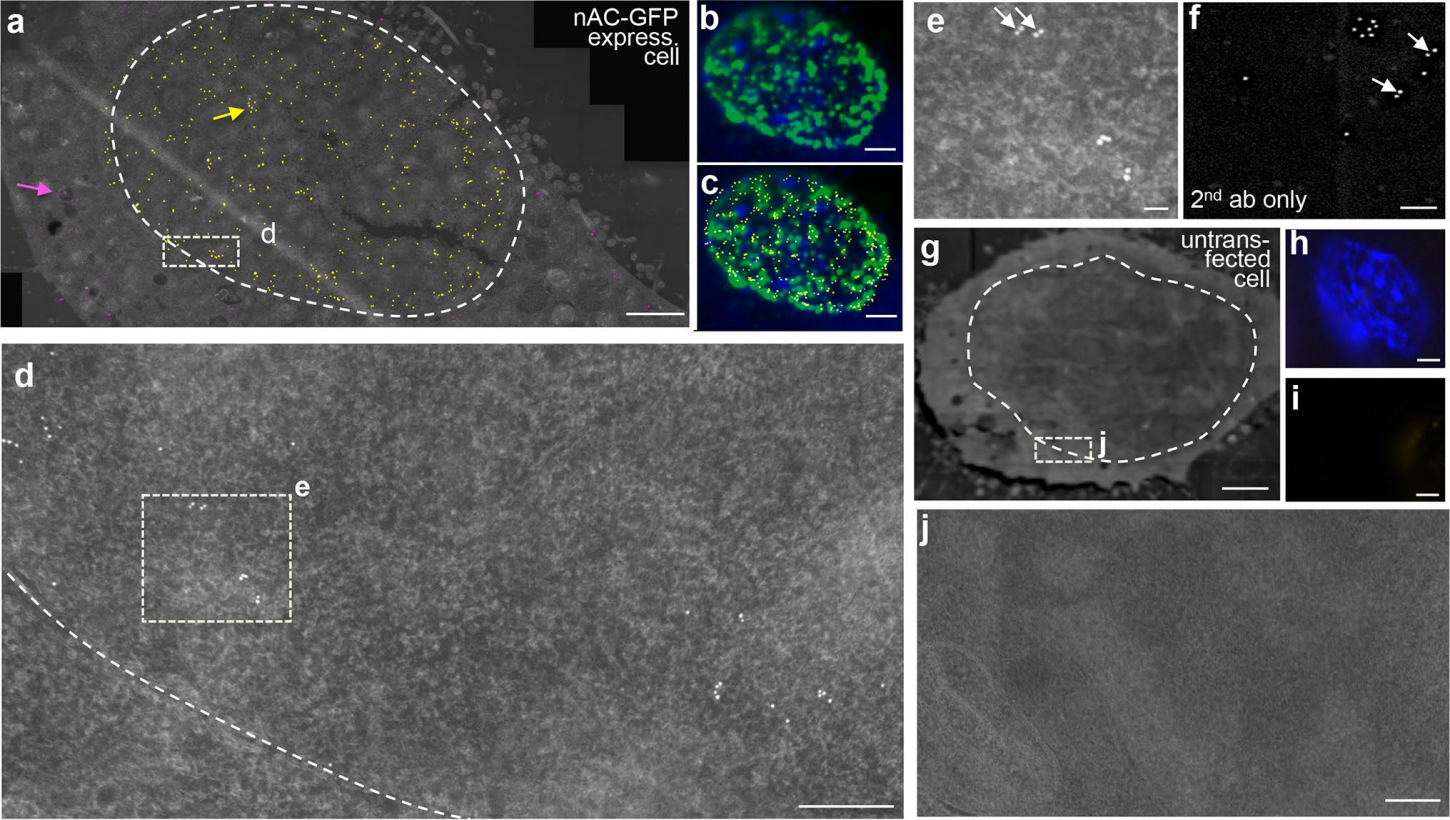
Localization and structure of endogenous nuclear actin in fibroblasts (NIH3T3 cells). **a** Overview of the distribution of all yellow false-colored gold particles labeling actin in a nuclear section of a cell expressing the nAC-GFP construct. The nAC-GFP construct recognizing actin gave signals predominantly in the nucleoplasm (surrounded by the dashed line) and only few signals were present in the cytoplasm (labeled in magenta). The composite image was derived from merging 72 single high-magnification pictures. Actin signals were spread all over the nucleoplasm with certain territories being spared (see **b**, **c**). **b** The same nucleus shown in (**a**) was labeled with fluorescent antibodies (green) recognizing the nAC-GFP and chromatin was stained with DAPI (blue). Actin signals were localized outside regions of prominent DAPI staining. **c** The picture in (**b**) was additionally co-localized with the yellow-colored gold particles. There was a high degree of co-localization of fluorescent and immunogold-labeled actin signals. **d**, **e** Higher magnifications of boxes depicted in (**a**) and (**d**). Gold particles were found as individual particles, in duplets and clusters of several particles. Many actin signals appeared to assemble in duplets [arrows in (**e**), but see (**f**) for a word of caution]. The dashed line in (**d**) labels the cytoplasmic-to-nuclear border. **f** Gold-labeled secondary antibodies accumulated on empty grids also in duplets (arrows in **f**) in the absence of incubation with first antibodies directed against GFP. This suggests nonspecific aggregation of secondary antibodies. **g**–**j** An untransfected cell not expressing the nAC-GFP construct was subjected to the same staining procedure as the cell in (**a**–**e**). No gold particles were visible suggesting the absence of nonspecific binding of the first and secondary antibodies (**g**, **j**). The cell was positive for DAPI (**h**) but did not show any GFP fluorescence (**i**). Scale-bars **a**–**c**, **g**–**i** = 2 μm; **d**, **j** = 200 nm; **e**, **f** = 50 nm

**Fig. 3:**
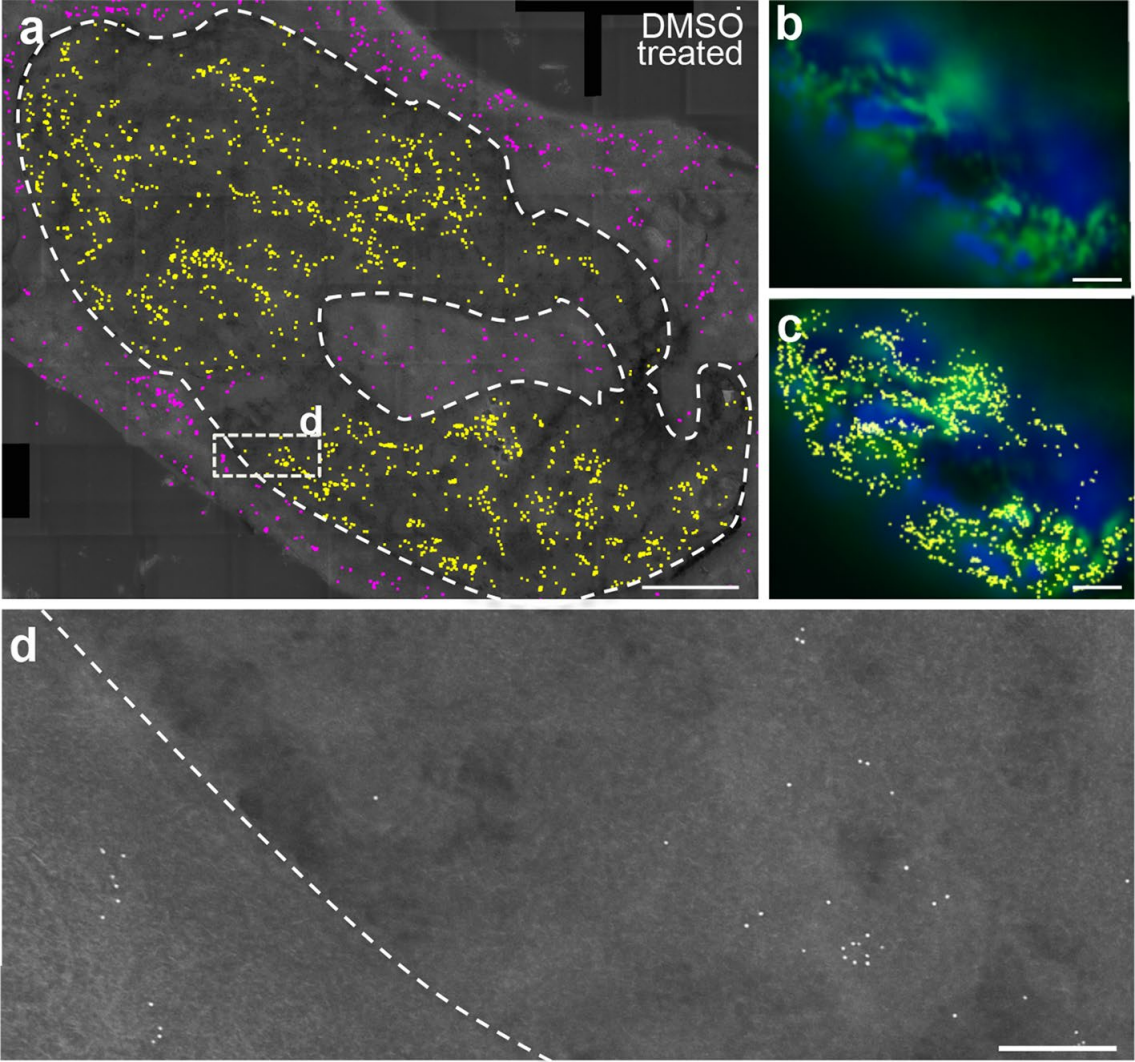
Analysis of nuclear actin localization in DMSO-treated NIH3T3 cells. **a** Overview of the distribution of yellow or magenta false-colored gold particles labeling actin in the nucleus and cytoplasm, respectively, of a DMSO-treated cell expressing the nAC-GFP construct. The nAC-GFP construct recognizing actin gave comparable signals in the nucleoplasm and cytoplasm. The dashed line marks the nuclear border. **b** The same nucleus shown in (**a**) was labeled with fluorescent antibodies (green) recognizing the nAC-GFP and chromatin was stained with DAPI (blue). **c** The picture in (**b**) was also co-localized with the yellow-colored gold particles. There was a high degree of co-localization of fluorescent and immunogold-labeled actin signals. **d** Higher magnifications of box depicted in (**a**). Gold particles were found as individual particles but also as clusters of several particles in both the cytoplasm and nucleus. The dashed line labels the cytoplasmic-to-nuclear border. Scale-bars **a**–**c** = 2 μm; **d** = 200 nm

**Fig. 4:**
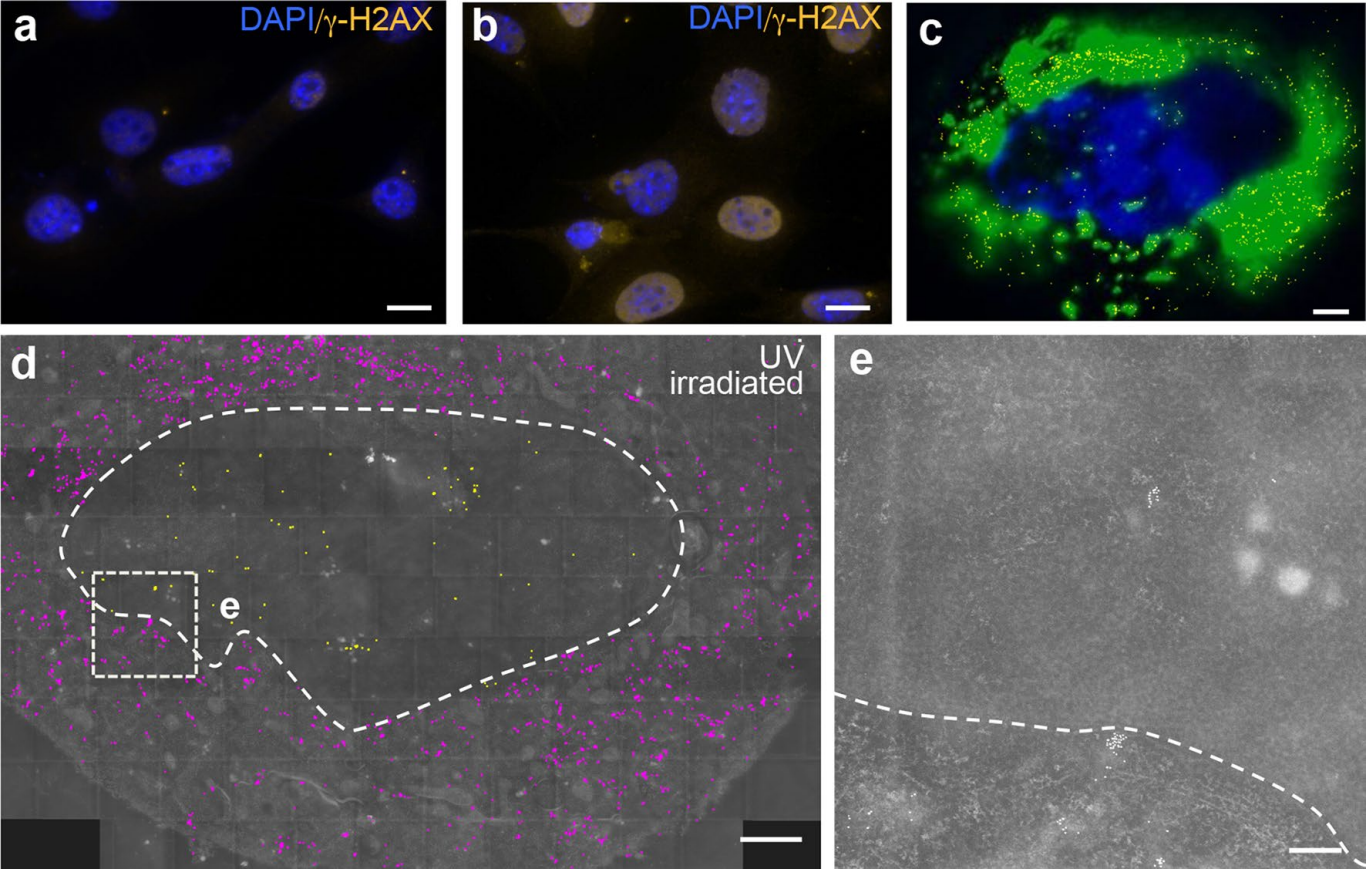
Localization and structure of endogenous nuclear actin in UV-irradiated NIH3T3 cells. **a**, **b** An untreated (**a**) and a UV-irradiated (**b**) cells were stained for DAPI (blue) and γ-H2AX (orange), labeling DNA double-strand breaks. UV irradiation enhanced DNA damage compared to untreated cells. **c** Co-localization of GFP fluorescence, DAPI and false-colored gold particles (yellow). There was a strong co-localization of fluorescent and immunogold-labeled actin signals. **d** A composite picture depicting the distribution of nuclear yellow or cytoplasmic magenta false-colored gold particles of an UV-irradiated cell expressing the nAC-GFP construct. More false-colored gold particles were observed in the cytoplasm compared to the nucleus. The dashed line marks the nuclear border. **e** Higher magnifications of box depicted in **d**. Many gold particles accumulated in clusters in both the cytoplasm and nucleus. The dashed line labels the cytoplasmic-to-nuclear border. Scale-bars **a**, **b** = 10 μm; **c**, **d** = 2 μm; **e** = 200 nm

In these un-stimulated cells analyzed by conventional fluorescent microscopy, approximately 70% of cells showed uniform nuclear actin distribution or small filaments (arrows, Fig. 1b). In the remaining 30% of cells we observed larger filaments. For simultaneous detection of endogenous actin by fluorescence and EM, we applied a first antibody directed against the GFP-tag of the nanobody (Fig. 1d). Subsequently, we used gold-conjugated and fluorescently labeled secondary antibodies (Fig. 1d). This protocol enabled us to identify actin-expressing cells by fluorescent microscopy before imaging in the electron microscope (Fig. 1e; red circles). Since additional gold-labeling was performed on the same section, we could analyze cells marked in the fluorescence microscope by EM (Fig. 1e). From each cell (*N* = 17 cells in total), we analyzed one single section through the nucleus. We took approximately 60–100 individual high-resolution EM pictures (grey frames in Fig. 1f) that were merged to one composite picture covering the entire area of the nucleus and parts of the neighboring cytoplasm within this section. To facilitate visualization of gold particles, we false-colored individual 6 nm gold particles with yellow dots of 90 nm (Fig. 1g; see also Figs. 2, 3, 4).

### Localization and structure of endogenous nuclear actin in fibroblasts

In all untreated control cells analyzed (*N* = 7 cells), we observed the presence of gold particles recognizing endogenous nuclear actin (false-colored in yellow) in the nucleus (Fig. 2a). On average, we quantified 700 gold particles (720 ± 295; *N* = 7 cells) on one 100-nm section taken through the nucleus of that cell. Please note that single yellow dots have a diameter of approximately 90 nm, thereby potentially covering several 6 nm gold particles. In the neighboring cytoplasm, only a few gold particles were observed (magenta dots in Fig. 2a). Thus, in agreement with the localization of the nanobodies by immunofluorescence (Fig. 2b, c), the majority of NLS-tagged chromobody molecules appeared to be efficiently transported to the nucleus where they can recognize actin molecules. This underscores the specificity of the NLS-tagged nanobody to recognize nuclear localized actin (Fig. 2a). The only few detectable gold particles in the cytoplasm of untreated cells also indicated almost no binding of secondary gold-labeled antibodies to nonspecific cellular proteins or structures (Fig. 2a). Overall, this suggests that endogenous nuclear actin can be specifically visualized by this EM approach.

**Fig. 5:**
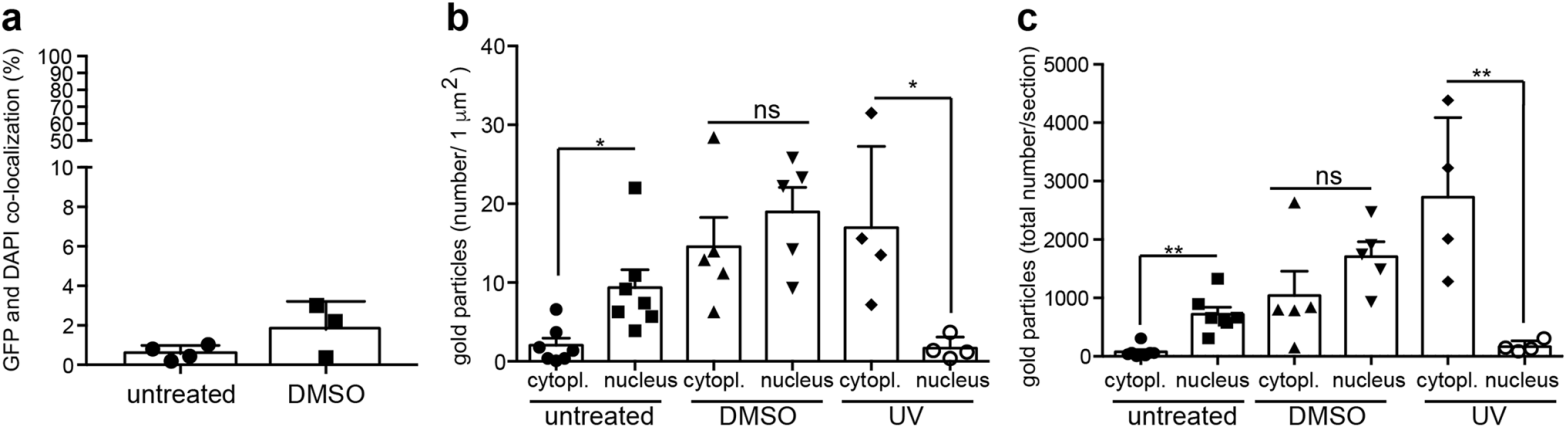
Quantification of co-localization and abundance of actin-associated gold particles. **a** Co-localization of fluorescent signals derived from GFP and DAPI in the nucleus of untreated and DMSO-treated cells. Only 1–2% of both signals co-localized and the majority of signals were complementary. **b**, **c** Quantification gold particles/μm^2^ (**b**) or total number of gold particles/section (**c**) for cytoplasm and nucleus for the different conditions indicated. In untreated cells, more gold particles were present in the nucleus. DMSO treatment enhanced abundance of gold particles in cytoplasm and nucleus. After UV irradiation, more gold particles were found in the cytoplasm. Each symbol in (**a**–**c**) reflects one section of one cell analyzed. *ns,* no significant difference, asterisks denote the degree of significant difference

We observed that all gold particles were spread almost throughout the entire nucleoplasm ranging from the peripheral lamina to the nucleus center (Fig. 2a). However, several regions in the nucleoplasm were devoid of actin signals (Fig. 2a–c). Since we co-stained the gold-labeled sections with fluorescent antibodies and DAPI, we can address a potential co-localization of actin signals with such chromatin regions (Fig. 2b, c). We noted that the majority of fluorescent actin signals (green Fig. 2b) was found outside those regions stained most intensively for DAPI (blue Fig. 2b). In fact, quantification revealed that 98% of all GFP and DAPI signals were not co-localized, while approximately 2% were co-localized (see Fig. 5a for quantification). When co-localizing the fluorescent (green) with the immunogold (yellow) signals we observed a high degree of co-localization of both signals (Fig. 2c). As seen for the fluorescent actin signals (Fig. 2b), also the gold-labeled actin proteins were largely absent from the DAPI-positive nuclear regions (Fig. 2c). Strong DAPI-positive regions within the nucleus are widely considered to consist mainly of heterochromatin that, in contrast to euchromatin, is more compact and less transcriptionally active (Fiserova et al. 2017; Schmid et al. 2017). Thus, although direct evidence is missing, our co-staining data suggest a preferential localization of actin in more transcriptionally active euchromatin. Previous studies have shown nuclear actin abundance in puncta surrounding DNA (Wineland et al. 2018), chromatin fibers (Milankov and De Boni 1993) and actin enrichment in structures reminiscent of transcription foci (Cruz and Moreno Diaz de la Espina 2009). However, actin localization in transcriptionally inactive nuclear regions was also described (Philimonenko et al. 2010). Such a localization of actin in transcriptionally active nuclear regions would be in line with the well-documented actin function in stimulating the general transcription machinery (Bajusz et al. 2018; Melak et al. 2017; Virtanen and Vartiainen 2017) and MRTF/SRF-dependent gene expression in particular (Vartiainen et al. 2007).

We were also interested to inspect whether nuclear actin molecules accumulate in specific structures or arrangements. Closer inspection of gold-labeled actin signals revealed single isolated gold particles and accumulations of individual gold signals in higher order arrangements such as duplets or clusters of several gold particles (Fig. 2d, e). Quantification showed that approximately 35% of all gold particles were isolated, i.e., not surrounded by a further particle within a distance of at least 30 nm (Fig. 2a, d, e). A high percentage (approx. 40%) of gold-labeled signals accumulated in duplet structures suggesting presence of actin molecules in duplets (Fig. 2d, arrows in Fig. 2e). However, here a word of caution is advised. When we provoked nonspecific labeling by omitting the first antibody and only applying secondary gold-conjugated antibodies to the Poly-l-lysine coated grid surface without subsequent washing steps, an identical percentage of duplets was observed (Fig. 2f). This suggests that 6 nm gold-labeled secondary antibodies tend to aggregate and form duplets or higher order structures, which could be misinterpreted as a specific protein arrangement. In cells not expressing the nAC-GFP construct but subjected to the identical staining procedure, as outlined in Fig. 1, we did not observe any gold particles (Fig. 2g–j). This further documents the specificity of the established protocol to stain for the localization of endogenously localized nuclear actin.

An obvious question is whether nuclear F-actin filaments were observed in this EM study. The nanobody recognizes actin proteins in monomeric G-actin as well as in polymerized F-actin (Melak et al. 2017). However, it is difficult to deduce the existence of actin filaments simply from accumulations of several actin-labeled gold particles, since those might reflect actin present in G- or F-actin. To draw this conclusion more firmly, we expected to observe long stretches of filaments decorated with many gold particles. Such actin filaments were observed upon nucleus-restricted overexpression of actin mutant proteins in our previous study (Kokai et al. 2014). However, in the present study, we did not directly observe such linear arrays of gold particles indicative of F-actin filaments on any analyzed section. It should be kept in mind that in the present study we analyzed endogenous nuclear actin levels which are clearly less abundant compared to our previous study utilizing actin overexpression. Therefore, it might be more difficult to observe such filamentous structures. Furthermore, we analyzed sections with a thickness of only 100 nm, thus it is highly unlikely to observe long microfilaments within the narrow thickness of such a thin section and only short stretches might be observable if any polymers at all. A final caveat is incomplete penetration of antibodies into the entire depth of such a LR gold-embedded section thereby limiting binding to and detection of epitopes.

### Analysis of nuclear actin localization upon administration of cell stress

Actin is known to form rod-like aggregates together with cofilin in the presence of cell stressing stimuli such as neurodegenerative proteins, DNA damage, heat stress or DMSO (Serebryannyy et al. 2016b).

To address the impact of cell stress on nuclear actin localization and structural organization, we applied DMSO to NIH3T3 cells (Fig. 3; *N* = 6 cells). DMSO application resulted in cell rounding indicative of stressful conditions. Gold particles were distributed over the entire nucleoplasm (Fig. 3a), but once again regions staining most strongly DAPI-positive were devoid of either fluorescent actin signals (green in Fig. 3b) or yellow-marked gold particles (Fig. 3c). Thus, as seen for unstressed cells (Fig. 2), nuclear actin and highly DAPI-positive chromatin—indicative of heterochromatin—were complementarily localized. Fluorescent and immunogold-labeled actin proteins co-localized strongly (Fig. 3c) as also seen for unstressed NIH3T3 cells (Fig. 2c).

In DMSO-treated cells, yellow-labeled gold particles were observed in the nucleoplasm, but gold particles were also, to a considerable extent, present in the cytoplasm (labeled in magenta in Fig. 3a). This is in contrast to unstressed cells, where only few gold particles were present in the cytoplasm (see Fig. 2a). In both the cytoplasm and nucleus we observed more gold particles/area in DMSO-treated cells (Fig. 3a) compared to unstressed cells. This also resulted in a twofold increase in total numbers of gold particles in the entire cell section including cytoplasm and nucleus (DMSO: 1488 ± 764 particles; unstressed cells: 720 ± 295 particles).

Beside DMSO, we applied UV irradiation to trigger DNA damage through induction of DNA double-strand breaks (Fig. 4; *N* = 4 cells). Indeed, *γ*-H2AX, a marker for DNA double-strand breaks, was upregulated upon UV irradiation compared to untreated cells (Fig. 4a, b). We noted in those stressed cells that GFP fluorescence derived from the nanobody was frequently pronounced in the cytoplasm rather than in the cell nucleus (Fig. 4c). As before (Figs. 2 and 3), there was a high co-localization of GFP fluorescence and false-colored gold particles (Fig. 4c). As noted with the localization of the GFP fluorescence (Fig. 4c), there were also more magenta false-colored gold particles in the cytoplasm of this UV-irradiated cell (Fig. 4d). In contrast, the abundance of yellow false-colored gold particles present in the nucleus was reduced (Fig. 4d). This was also confirmed when quantifying the number of gold particles/area (Fig. 5b) as well as total cytoplasmic or nuclear numbers of gold particles/section (Fig. 5c) in several cells. Inspection of gold particles at higher magnification revealed clustering of many gold particles (> 10) at cytoplasmic or nuclear positions (Fig. 4e). Finally, we also quantified the co-localization of GFP and DAPI signals (depicted in Figs. 2b and 3b) in Fig. 5a, as well as the subcellular localization of gold particles (Fig. 5b, c) in unstressed (Fig. 2), DMSO-treated (Fig. 3) and UV-irradiated (Fig. 4) cells.

Thus, in summary, two different types of cell stressors, DMSO (Fig. 3) and UV irradiation (Fig. 4), altered the sub-cellular abundance of actin proteins in the cells by enhancing the numbers of gold-associated actin signals in the cytoplasm. Mechanistically, this might involve a direct upregulation of actin protein production by application of cell stress. In addition, cell stress could modulate the G- vs. F-actin ratio and thereby also alter the detection by nanobodies in such way that more actin proteins can be visualized. Finally, it has to be taken into account that cell stress might impinge on the integrity of the cells, particularly the nuclear envelope permeability and thereby nucleocytoplasmic shuttling of proteins which might affect the abundance of actin proteins in those two compartments.

As described above for unstressed cells, we also did not observe actin filaments or large clusters of gold particles reminiscent of stress-induced aggregates in DMSO or UV-treated cells (taken together, ten sections from ten different cells). This finding was somehow surprising given that cell stress is known to induce actin/cofilin aggregation throughout the nucleus at least on the light-microscopical level (Belin et al. 2015; Serebryannyy et al. 2016b). However, as previously reported, such aggregates stain much stronger for cofilin than for phalloidin (Serebryannyy et al. 2016b) indicating more difficulties when attempting to stain for endogenous nuclear actin in such aggregates. In addition, such aggregates were best described in neurons and not fibroblasts used in this study. These restrictions together with the low abundance of endogenous nuclear actin and the low thickness of the sections (see discussion to Fig. 2, above) might impede on the visualization of actin filaments by electron microscopy.

### Outlook

In this study, we provide a novel CLEM approach allowing for the detection of nuclear actin by a chromobody-based detection protocol. However, our detection approach involves several antibody incubation steps for detecting the chromobody (see Fig. 1). Thus, in future studies it would be preferable to fuse the chromobody with an enzymatic tag, for example the APEX (ascorbate peroxidase) tag. In previous studies the APEX enzyme has been genetically fused to several proteins of interest (also including nanobodies). Subsequently, the fusion construct was expressed inside the cells and was successfully analyzed by electron microscopy (Lam et al. 2015; Ariotti et al. 2018). This would allow for a single-step detection reaction procedure (rather than multi-step used in our study) by directly fixing cells, followed by DAB (diaminobenzidine) substrate incubation and recruitment of electron-dense osmium providing EM contrast.

## Electronic supplementary material

Below is the link to the electronic supplementary material.

Supplementary material 1 (TIFF 175338 kb)

Supplementary material 2 (TIFF 226257 kb)

Supplementary material 3 (TIFF 304794 kb)
